# Retrospective observational study of transcatheter arterial chemoembolization (TACE) combined with PD-1 inhibitor and tyrosine kinase inhibitor (TKI) in advanced hepatocellular carcinoma

**DOI:** 10.1097/MD.0000000000050965

**Published:** 2026-10-16

**Authors:** Guohui Xu, Jianlong Zhang, Xiaojun Li, Shilan Fan, Xiao Wu, Cuncai Zhou

**Affiliations:** a Department of Hepatobiliary Surgery and Interventional Radiology, Jiangxi Cancer Hospital , Nanchang, Jiangxi, China.

**Keywords:** advanced hepatocellular carcinoma, overall survival, PD-1, transcatheter arterial chemoembolization, tyrosine kinase inhibitor

## Abstract

Transcatheter arterial chemoembolization (TACE) induces tumor necrosis and antigen release, but the resulting hypoxic microenvironment upregulates programmed death-ligand 1and vascular endothelial growth factor, providing a biological rationale for adding a programmed death-1 (PD-1) inhibitor and a TKI. The study aims to assess the effectiveness and safety of a treatment regimen consisting of TACE, tyrosine kinase inhibitors, and PD-1 inhibitors in patients diagnosed with advanced hepatocellular carcinoma (HCC) that cannot be treated surgically. This retrospective observational study (Jiangxi Cancer Hospital, Jan 2021–Jun 2023) enrolled 52 advanced HCC patients, with 45 included in final analysis. Primary endpoints were progression-free survival (PFS) and overall survival (OS); secondary endpoints covered objective response rate, Disease Association for the Study of the Liver and safety. Survival was analyzed via Kaplan–Meier method and Cox proportional hazards regression. The cohort comprised 38 men and 7 women with a mean age of 50.9 ± 12.06 years, and 36 patients (80.0%) were hepatitis B virus antigen positive. At 6 months, the PFS rate stood at 71.1%, while at twelve months, it was recorded at 51.7%. The median PFS reached 12.4 months (95% confidence interval: 9.8–15.0), while the median OS was found to be 24.2 months (95% confidence interval: 20.1–28.3). The objective response rate was 44.4% and Disease Association for the Study of the Liver was 91.1%. Barcelona Clinic Liver Cancer (BCLC) staging significantly affected prognosis, with median PFS for BCLC A, B, C, and D being 19.2, 16.5, 12.1, and 4.8 months, respectively (log-rank *P* = .016). Median PFS for first-, second-, and third-line treatments were 17.1, 12.6, and 3.2 months, respectively (log-rank *P* < .001). Patients with positive node expression had significantly shorter PFS (5.1 vs 9.9 months, HR = 2.89, *P* = .005) and OS (14.9 vs 25.0 months, HR = 3.24, *P* = .002) compared to those with negative node expression. No grade 5 adverse events occurred. TACE combined with tyrosine kinase inhibitor and PD-1inhibitors shows promising efficacy and acceptable safety for advanced HCC. BCLC staging, treatment line and node status significantly affect outcomes, with earlier intervention yielding greater survival benefit. These exploratory findings from a small retrospective single-arm study require confirmation in larger prospective trials.

## 1. Introduction

Hepatocellular carcinoma (HCC), also referred to as hepatoma, represents the most prevalent form of primary liver cancer. More than half of all newly diagnosed cases and deaths worldwide occur in China. The total incidence of hepatoma in China stands at 9.59%, ranking fourth in the incidence rate of malignant tumors and third in the mortality rate.^[1]^ Globally, liver cancer was the sixth most commonly diagnosed malignancy and the third leading cause of cancer death in 2022, accounting for approximately 866,000 new cases and 759,000 deaths.^[2]^ The global burden is projected to increase by more than 55% by 2040, driven by population aging and by the growing contribution of metabolic and alcohol related liver disease.^[3]^ Despite the approval of several systemic regimens, most patients are diagnosed at an unresectable stage, median survival rarely exceeds 2 years, and no validated biomarker guides the choice among available options, which leaves a substantial unmet clinical need.^[4]^

Chemotherapy and embolization therapy can lead to necrosis of tumor tissues, thereby reducing the release of immunosuppressive factors and weakening the inhibitory effect on immune function. Moreover, embolization therapy reduces the number of peripheral blood T cells and weakens the antitumor immune response.^[5]^ Additionally, necrotic tumor tissues activate the immune response by altering the phenotype of peripheral blood immune cells.^[6]^ After transcatheter arterial chemoembolization (TACE) treatment, distal blood supply deficiency creates a local hypoxic microenvironment, and expression of the transcription factor hypoxia-inducible factor-1α is upregulated. This leads to promoting the programmed death-ligand 1 (PD-L1) expression by acting on its promoter through the activation of signal transducer and activator of transcription-3, ultimately leading to immunosuppressive effects.^[7]^ Therefore, TACE and immune checkpoint inhibitors (ICIs) may have a synergistic effect. Using a combined strategy of immunology and targeted therapy leads to reprogramming the immune microenvironment, which in turn increases CD8 + cytotoxic T cell infiltration and activation and reduces the count of immunosuppressive T regulatory cells in hepatoma tissues. Therefore, double-blocking therapy of programmed death-1 (PD-1) and vascular endothelial growth factor receptor-2 (VEGFR-2) in hepatoma can promote vascular normalization and enhance antitumor immune response.^[8]^ The results of clinical trials, including SHARP,^[9]^ REFLECT,^[10]^ CheckMate040,^[11]^ and IMBrave150,^[12]^ showed that ICIs and tyrosine kinase inhibitors (TKIs) significantly improved the treatment of unresectable HCC. TACE has both positive and negative impacts on the tumor microenvironment. The positive effects specifically refer to the induction of tumor cell apoptosis by chemotherapy drugs during TACE treatment and the activation of T cell responses. Ischemia and hypoxia caused by embolization affect tumor DNA synthesis. However, TACE treatment has some limitations. TACE embolization leads to local tissue hypoxia, further inducing increased PD-L1expression in tumors. Meanwhile, tissue hypoxia may lead to elevated vascular endothelial growth factor expression levels in tumor tissue, facilitating angiogenesis in malignant tissues.^[13]^

The current study investigated the effects of TACE + TKIs + PD-1 inhibitor on progression-free survival (PFS) and overall survival (OS) in patients with advanced unresectable hepatoma, stratified by Barcelona Clinic Liver Cancer (BCLC), hepatitis B virus (HBV), treatment line, and extrahepatic metastasis. Adverse events (AEs) were also evaluated.

## 2. Materials and methods

### 2.1. Study design

This was a retrospective observational study conducted at Jiangxi Cancer Hospital. The study protocol was approved by the Ethics Committee of Jiangxi Cancer Hospital (2020ky080) and was funded by the Jiangxi Provincial Health and Science and Technology Plan (SKJP220201578).

### 2.2. Subjects

Patients with advanced unresectable hepatoma who were treated with TACE + TKIs + PD-1 inhibitor at Jiangxi Cancer Hospital from January 2021 to June 2023 were enrolled. Written informed consent was obtained. Treatment line was defined by the number of systemic regimens received before the start of TACE + TKI + PD-1 inhibitor therapy: first line, no prior systemic therapy; second line, one prior systemic regimen; third line, 2 or more prior systemic regimens. Prior locoregional therapy alone was not counted as a line of systemic treatment. Patients were diagnosed with HCC per the European Association for the Study of the Liver clinical practice guidelines, based on dynamic computed tomography (CT) or dynamic magnetic resonance imaging (MRI) results.^[14]^ Subjects must meet all of the following entry criteria to be eligible for this study: Age: 18 to 75 years old, acceptable for both men and women; Patients diagnosed by clinical or histological pathology, and presenting with a minimum of 1 measurable lesion as defined by the modified Response Evaluation Criteria in Solid Tumors (mRECIST) criteria; The TACE +immune + anti-angiogenic targeted drugs are required for treatment; No other systemic malignancies are combined; Expected survival period of >12 weeks; Full organ reserve function: Hemoglobin 90g/L, neutrophils 1.510^*9*^*/L,* platelet *50*10^9^/L, Total Bilirubin <2 mg/dL, Serum Albumin 28g/L, Aspartate aminotransferase, alanine aminotransferase <5 times normal limits, normal limits of blood urea nitrogen and creatinine <1.5 times; Women of childbearing age and men with partners of childbearing age need to use contraception according to the protocol within 120 days after signing the informed drug to the last medication; All participants voluntarily enrolled in the study provided written informed consent, demonstrated strong compliance, and actively participated in follow-up assessments; and the investigator considers a benefit.

Subjects will not be enrolled in this study for any of the following conditions: Any episode of digestive system bleeding reported in the 6 months leading up to the first drug administration; Uncontrolled pleural effusion, pericardial effusion, or ascites are present, necessitating repeated drainage procedures; Previous history of allergy to immunotherapy and anti-vascular targeted drug components; Several conditions may interfere with oral drug administration, including difficulty swallowing, nausea, vomiting, persistent diarrhea, and intestinal obstruction; and Patients were not eligible if they received any of the following: A. Use of another investigational medication within the 4 weeks preceding the first dose of the study drug; B. Requirement for systemic corticosteroids exceeding 10 mg of prednisone (or its equivalent) or other immunosuppressive agents within the 2 weeks before starting the study drug, except local esophageal inflammation, allergy prevention, or use for managing nausea/vomiting. Exceptions may be discussed with the sponsor. Inhaled/topical corticosteroids and adrenal replacement therapies were allowed at doses ≤10 mg of prednisone per day, provided there was no active autoimmune disease; C. Vaccination with an antitumor or live vaccine within 4 weeks before the study drug is first given; D. Simultaneous participation in another clinical study, unless it is non-interventional or in its follow-up stage; 6. Patients with a history of active autoimmune diseases and autoimmune diseases (e.g. interstitial pneumonia, colitis, hepatitis, hypophysitis, vasculitis, nephritis, including but not limited to these diseases or syndromes); except for vitiligo or recovered childhood asthma/allergy without any intervention in adults; autoimmune-mediated hypothyroidism treated with a stable dose of thyroid replacement hormone; type I diabetes with a stable dose of insulin; 7. A previous immunodeficiency history, including a positive HIV test, other congenital or acquired immunodeficiencies, or a previous allogeneic bone marrow transplantation and organ transplantation; 8. Presence of any severe infection, classified above Grade 2 by Common Terminology Criteria for Adverse Events, within 4 weeks of the first study drug dose. Examples include severe pneumonia, bacteremia, or complications requiring inpatient care. Active lung inflammation seen on baseline imaging, infection-related symptoms or signs within 2 weeks before the study drug initiation, or the need for oral/IV antibiotics (excluding prophylactic use) were also grounds for exclusion; 9. Patients diagnosed with another malignancy within 5 years before enrollment, pregnant or lactating women, individuals with serious health conditions (e.g., psychiatric illness), markedly abnormal labs, or personal/social barriers that could interfere with data collection were excluded at the investigator’s discretion.

### 2.3. TACE

The tumor supply artery was super-selected during TACE, followed by the administration of a mixture containing chemotherapy agents and iodine oil. Alternatively, polyvinyl alcohol particles were used to embolize the tumor supply artery until the embolic agents were completely stagnated in the tumor blood vessels. The TACE treatment was implemented using “on-demand TACE” based on the imaging examination results.

The TACE plan was repeated in the following situations: incomplete tumor necrosis; lesion regrowth; and new lesions appear. Repeat TACE was decided at each scheduled imaging visit and was performed at a minimum interval of 6 weeks from the preceding session, provided that liver function remained Child-Pugh A or B and no uncontrolled adverse event was present.

### 2.4. TKIs + PD-1 inhibitor

PD-1 inhibitor: Camrelizumab (specification: 200 mg): 3mg/kg, Sintilimab (specification: 10 ml:100 mg): 200 mg; administered once every 3 weeks, with 1 treatment cycle lasting 3 weeks. Injection immunotherapy administration was completed 4 to 10 days after the first TACE treatment. The administration was given according to the instructions/indication dosage, administered every 3 weeks. When the disease progressed or toxicity was intolerant, novel antitumor treatments were initiated, and withdrawal was informed or the researcher determined that the subject needed to withdraw from the study. The maximum duration of immunotherapy was 2 years.

TKIs: Sorafenib (Specification: 0.2 g 60 tablets): Oral administration, 400 mg each time, twice daily; Donafenib (Specification: 0.1 g 40 tablets): Oral administration, 0.2 g each time, twice daily. Injection immunotherapy administration was completed 7 to 10 days after the first TACE treatment. For patients experiencing adverse reactions to anti-angiogenic targeted drugs, adjust the dosage based on specific conditions without reverting to previous doses. Treatment continues until disease progression or intolerable toxicity occurs.

### 2.5. Efficacy evaluation

During and after treatment, the effectiveness of the treatment was evaluated by dynamic MRI or dynamic CT every 6 to 8 weeks. Treatment efficacy was assessed using the mRECIST.^[15]^ Contrast enhanced CT or MRI was obtained at baseline, every 6 to 8 weeks during the first 12 months, and every 10 to 12 weeks thereafter, and each examination was reviewed independently by 2 radiologists, with disagreements resolved by consensus. The duration of PFS was defined as the time interval between the commencement of initial treatment and the occurrence of disease progression. The duration of OS was measured from the point of study enrollment until the occurrence of death, regardless of cause. All treatment-related AEs were determined according to the Common Terminology Criteria for Adverse Events (version 5.0) proposed by the National Cancer Institute. The follow-up period involved conducting survival follow-ups every 2 months after disease progression or withdrawal from the group until the patient’s death.

### 2.6. Data collection

The collected data included demographic, clinical, and laboratory data, hepatitis B surface antigen status, alpha-fetoprotein level, BCLC staging,^[16]^ extrahepatic metastasis, node expression, and treatment line.

### 2.7. Statistical analysis

All statistical analyses were performed using SPSS software (version 26.0, IBM Corp., Armonk). Continuous variables were expressed as mean±standard deviation and compared using the independent samples t-test after confirming normal distribution by the Shapiro–Wilk test. Categorical variables were presented as frequencies and percentages and compared using the chi-square test or Fisher’s exact test, as appropriate. The Kaplan–Meier method was employed to assess the median PFS, median OS, duration of remission, and the overall 95% confidence interval (CI), followed by the generation of a survival curve. The log-rank test was used to compare survival curves between subgroups. Univariate Cox proportional hazards regression analysis was performed to calculate hazard ratios with 95% CIs for prognostic factors. Point estimation was performed on efficacy endpoints, including objective response rate (ORR) and disease control rate Disease Association for the Study of the Liver (DCR), followed by obtaining the overall 95% CI. Pearson correlation analysis was used to assess the linear relationship between variables. Descriptive analysis was performed on other secondary efficacy indicators. Patients with incomplete follow-up data were excluded from the final analysis. No imputation was performed. Because data were not assumed to be missing completely at random, this complete case approach may introduce attrition bias, and the estimates reported here should be interpreted with this limitation in mind. A two-sided *P* value <.05 was considered statistically significant.

## 3. Results

### 3.1. Characteristics

The study included 52 patients treated with the combination of TACE, TKIs, and PD-1 inhibitors. Follow-up was not possible for 5 patients, and 2 others had insufficient data, leaving 45 patients eligible for final analysis (Fig. 1). The cohort consisted of 38 males and 7 females, with a mean age of 50.9 ± 12.06 years. Among them, 36 (80.0%) had a positive HBV antigen status, 32 (71.1%) were diagnosed with BCLC C, 14 (31.1%) had extrahepatic metastasis, 12 (26.7%) exhibited positive node expression, and 20 (44.4%), 13 (28.9%) and 12 (26.7%) received the regimen as first-, second- and third-line therapy, respectively (Table 1).

**Table 1:** Baseline characteristics of the analyzed cohort (n = 45).

Variable	Category	n (%) or mean±SD
Sex	Male	38 (84.4)
	Female	7 (15.6)
Age, yr		50.9 ± 12.06
HBV status	Positive	36 (80.0)
	Negative	9 (20.0)
BCLC stage	A	3 (6.7)
	B	5 (11.1)
	C	32 (71.1)
	D	5 (11.1)
Extrahepatic metastasis	Yes	14 (31.1)
	No	31 (68.9)
Node expression	Positive	12 (26.7)
	Negative	33 (73.3)
Treatment line	First–line	20 (44.4)
	Second–line	13 (28.9)
	Third–line	12 (26.7)

Percentages are based on the 45 patients in the final analysis and may not sum to 100 because of rounding.

BCLC = Barcelona Clinic Liver Cancer, HBV = hepatitis B virus, SD = standard deviation.

**Figure 1.**
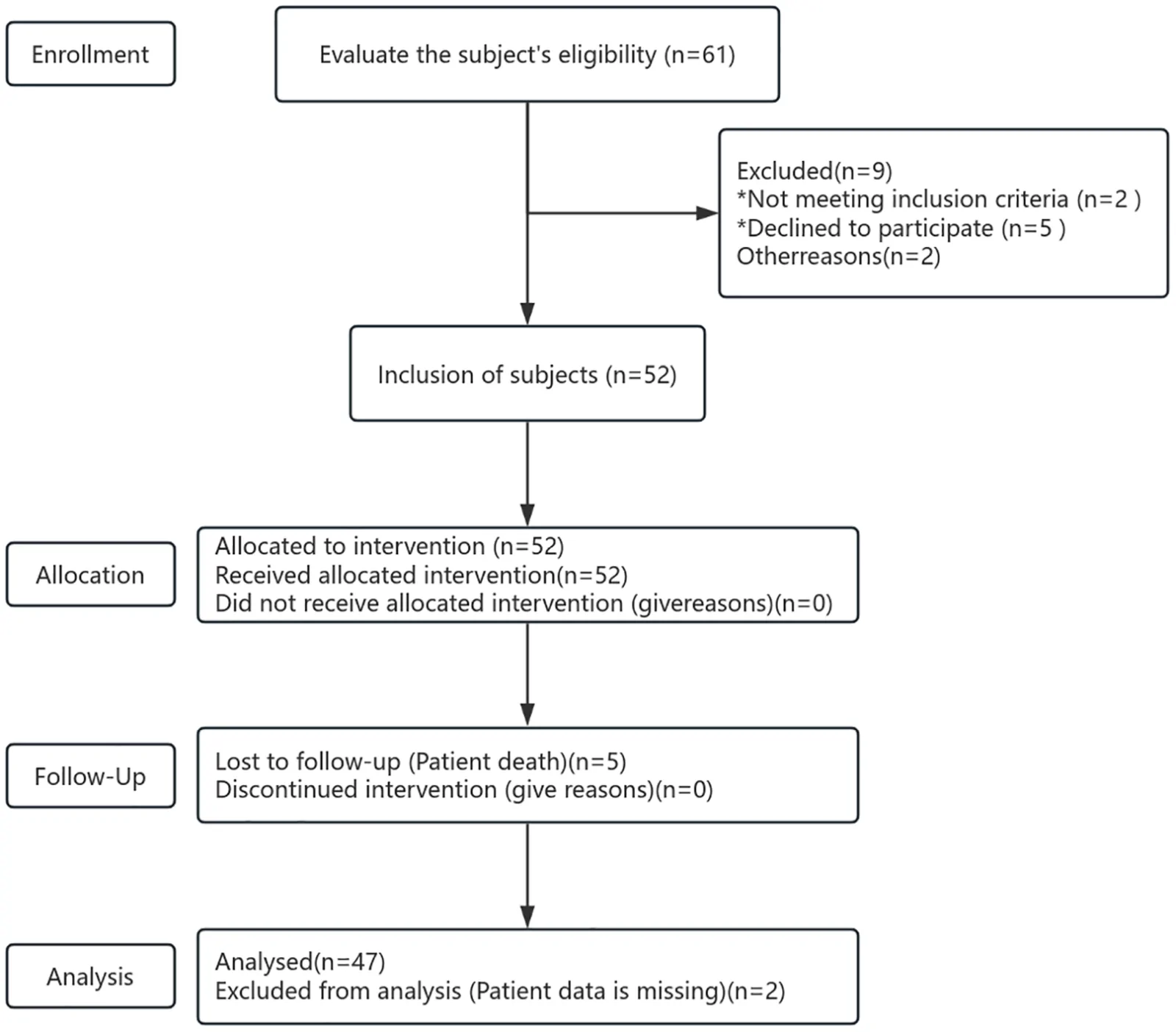
CONSORT flow diagram of patient enrollment, allocation, follow-up, and analysis in the retrospective study of transcatheter arterial chemoembolization (TACE) combined with programmed death-1 (PD-1) inhibitor and tyrosine kinase inhibitor (TKI) for advanced hepatocellular carcinoma. Of 61 patients screened, 9 were excluded, 52 were treated, 5 were lost to follow-up because of death, and 2 were excluded for missing data, leaving 45 patients in the final analysis. PD-1 = programmed death-1, TACE = transcatheter arterial chemoembolization, TKIs = Tyrosine Kinase Inhibitors.

### 3.2. Treatment results

The PFS rate was 71.1% at 6 months and 51.7% at 12 months. Median values for PFS and OS were 12.4 months (95% CI: 9.8–15.0 months) and 24.2 months (95% CI: 20.1–28.3 months), respectively. Evaluations using mRECIST showed complete responses in 4 patients (8.9%), partial response in 16 (35.5%), stable disease in 21 (46.7%), progressive disease in 4 (8.9%), yielding an ORR of 44.4% (95% CI: 29.6%–59.2%) and DCR of 91.1% (95% CI: 82.8%–99.4%) (Figs. 2 and 3). In examining the correlation between PFS and OS, this study employs a scatter plot model for analysis. The results indicate that the scatter points generally exhibit an upward trend from the lower left to the upper right, suggesting that as PFS values increase, OS values also tend to rise, demonstrating a positive correlation between the two (*R* = 0.85, *P* < .001) (Fig. 4).

**Figure 2.**
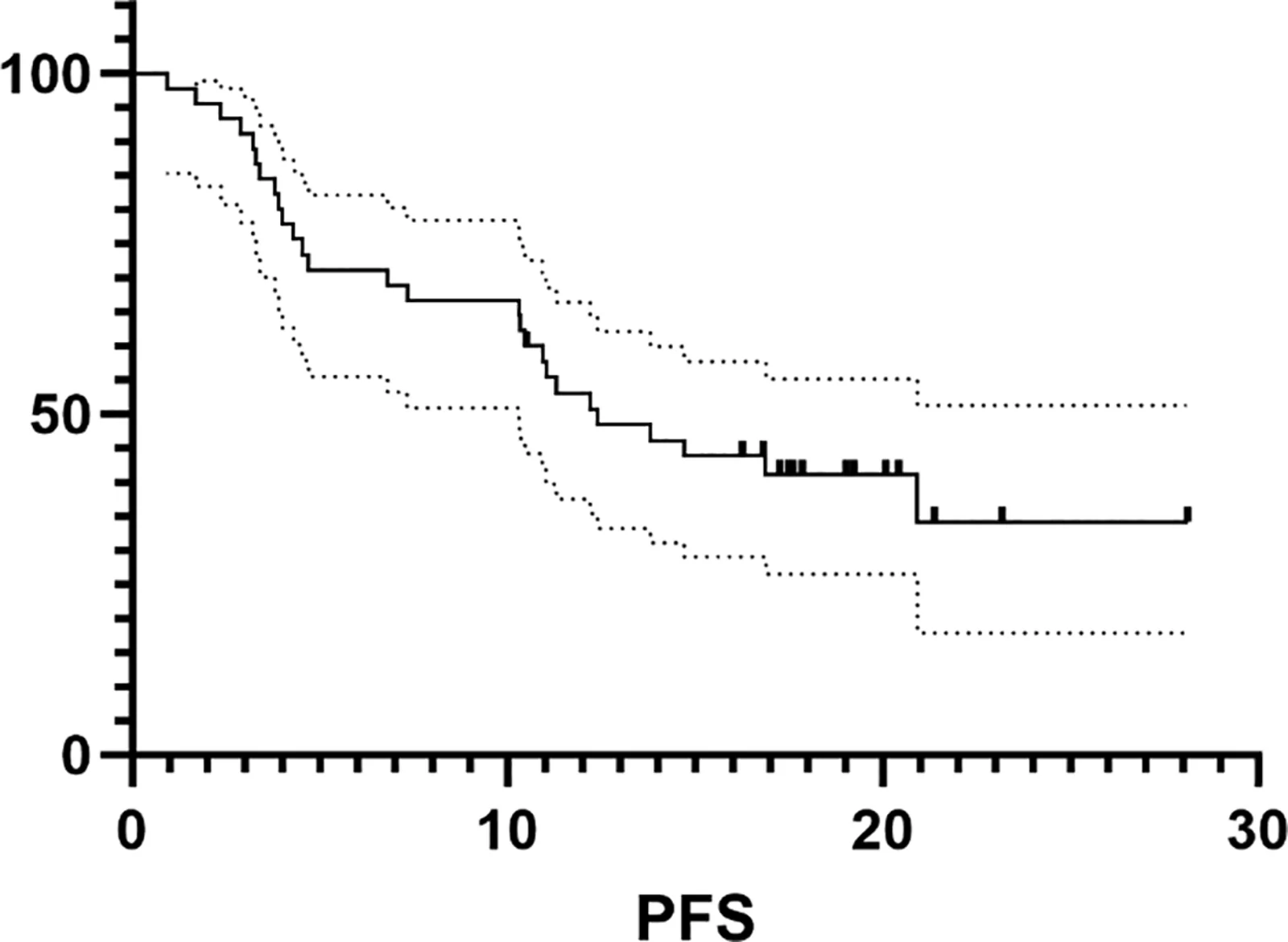
The K-M curve image of PFS. Kaplan–Meier curve showing progression-free survival (PFS) of patients with advanced hepatocellular carcinoma treated with TACE + PD-1 inhibitor + TKI. The x-axis shows time from the start of treatment in months and the y-axis the proportion of patients free of progression (%). The solid line is the Kaplan–Meier estimate, dotted lines the 95% confidence interval, and vertical ticks censored observations. Median PFS was 12.4 months (95% CI: 9.8-15.0); n = 45. CI = confidence interval , PFS = Progression-Free Survival, TACE = transcatheter arterial chemoembolization, TKIs = Tyrosine Kinase Inhibitors.

### 3.3. Stratified analysis

Multiple univariate analyses were conducted based on baseline characteristics. Correlation analysis was employed to measure the linear association strength between variables, with the core outputs being the *R*-value (range: −1 – 1) and the *P* value, including the HBV status, BCLC staging,^[13]^ extrahepatic metastasis status, node expression, and treatment line.

No statistically significant variations were detected across gender, age, or HBV infection status (*P* = .24, 0.79, and 0.66, respectively). Meanwhile, BCLC staging, treatment line, extrahepatic metastasis, and node expression were statistically significantly different (*P* = .016, *P* < .001, *P* = .015, and *P* = .001, respectively) (Table 2). Correlation analysis through a multi-factor heatmap (Fig. 5) displays the correlation coefficient matrix between multiple variables, intuitively presenting the strength of correlation through color gradients: dark blue indicates strong negative correlation (r approaching −1), while dark red indicates strong positive correlation (*r* approaching 1). Clinically, these findings suggest that treatment line number (*r* = −0.739) and node expression status (*r* = −0.462) are the strongest predictors of treatment response, indicating that earlier intervention and absence of lymph node involvement are associated with better outcomes. The conclusion demonstrates that PFS is highly synchronized with OS: patients with prolonged PFS typically also exhibit prolonged OS. Because these coefficients describe association rather than causation, they should be read as hypothesis generating signals for patient selection, and the strong PFS-OS correlation (*R* = 0.95) supports the use of PFS as an early surrogate readout in this setting rather than as an independent efficacy claim. The reformatted correlation results, with variables in rows and coefficients, CI and *P* values in columns, are presented in Table 2 and Table 3.

**Table 2:** Univariate correlation of baseline variables with treatment outcome.

Variable	*r*	95% CI	*P* value
Gender	0.178	−0.122 – 0.448	.241
Age	0.040	−0.257 – 0.330	.795
HBV status	−0.067	−0.354 – 0.231	.661
BCLC stage	−0.357	−0.589 to −0.071	.016
Treatment line	−0.739	−0.849 – −0.569	<.001
Extrahepatic metastasis	−0.360	−0.591 – −0.074	.015
Node expression	−0.462	−0.665 – −0.195	.001

A negative *r* indicates that a higher category value is associated with shorter survival. *P* values are 2-sided; values below .001 are reported as <.001.

BCLC = Barcelona Clinic Liver Cancer, CI = confidence interval, HBV = hepatitis B virus. *r*, Pearson correlation coefficient.

**Table 3:** Correlation of clinical variables with PFS and OS, with clinical interpretation.

Variable	*r* with PFS	*r* with OS	Clinical interpretation
Number of prior treatment lines	−0.74	−0.72	Strongest inverse association; supports earlier use of the regimen
Node expression (lymph node involvement)	−0.46	−0.42	Marker of more aggressive biology and reduced locoregional benefit
BCLC stage	−0.36	−0.54	Advanced stage and liver decompensation limit tolerability and benefit
Extrahepatic metastasis	−0.36	−0.29	Disease outside the liver limits the value of local control by TACE
Gender	0.18	0.16	No clinically meaningful association
Age	0.04	0.10	No clinically meaningful association
HBV status	−0.07	−0.01	No clinically meaningful association
PFS vs OS	1.00	0.95	PFS closely tracks OS and can serve as an early readout

BCLC = Barcelona Clinic Liver Cancer, HBV = hepatitis B virus, OS = overall survival, PFS = progression-free survival, TACE = transcatheter arterial chemoembolization. *r*, Pearson correlation coefficient; coefficients describe association, not causation.

**Figure 3.**
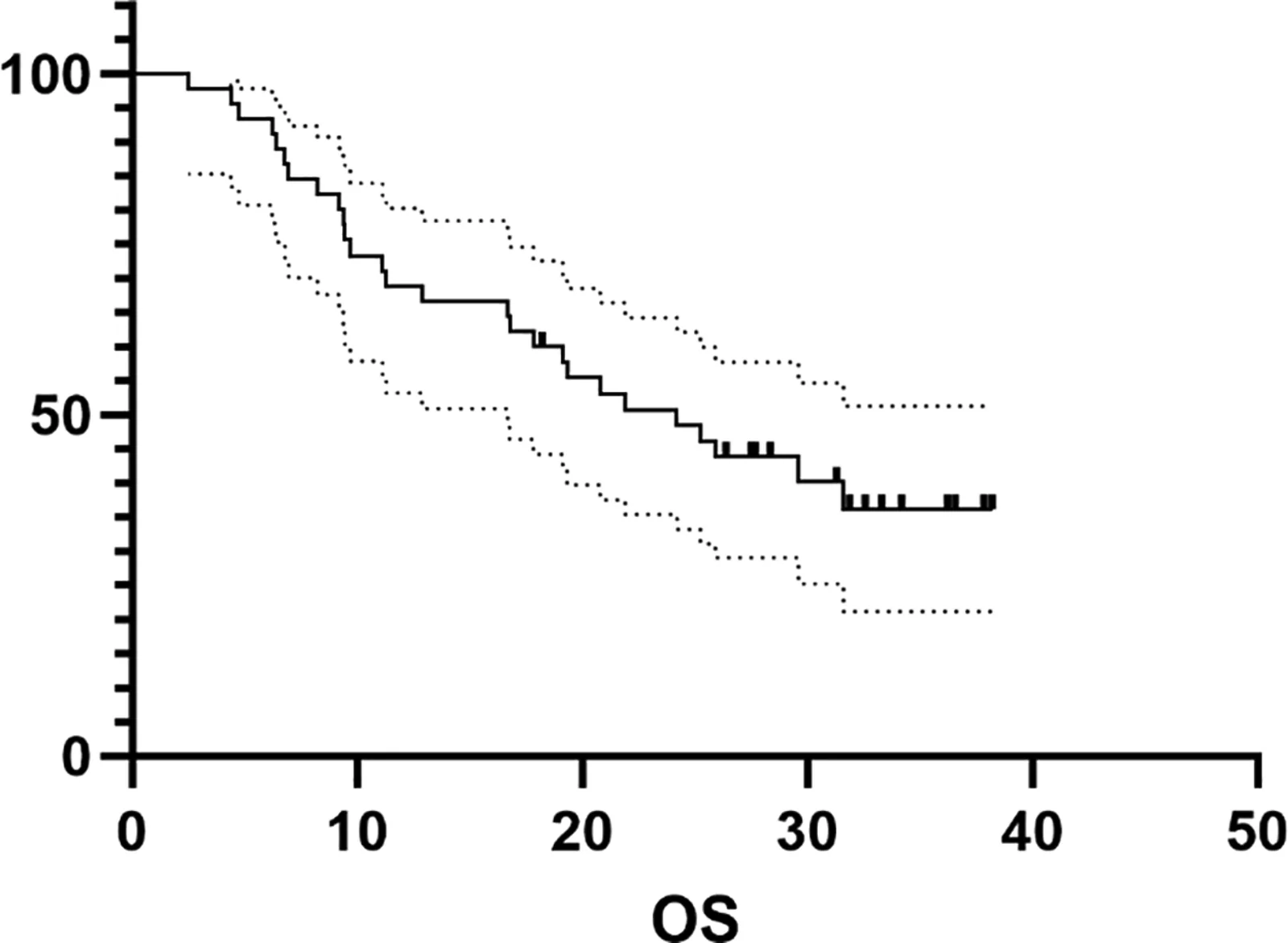
The K-M curve image of OS. Kaplan–Meier curve showing overall survival (OS) of patients with advanced hepatocellular carcinoma treated with TACE+PD-1 inhibitor+TKI. The x-axis shows time from enrollment in months and the y-axis the proportion of patients alive (%). The solid line is the Kaplan–Meier estimate, dotted lines the 95% confidence interval, and vertical ticks censored observations. Median OS was 24.2 months (95% CI: 20.1–28.3); n = 45. OS = Overall Survival, PD-1 = programmed death-1,TACE = transcatheter arterial chemoembolization, TKIs = Tyrosine Kinase Inhibitors.

**Figure 4.**
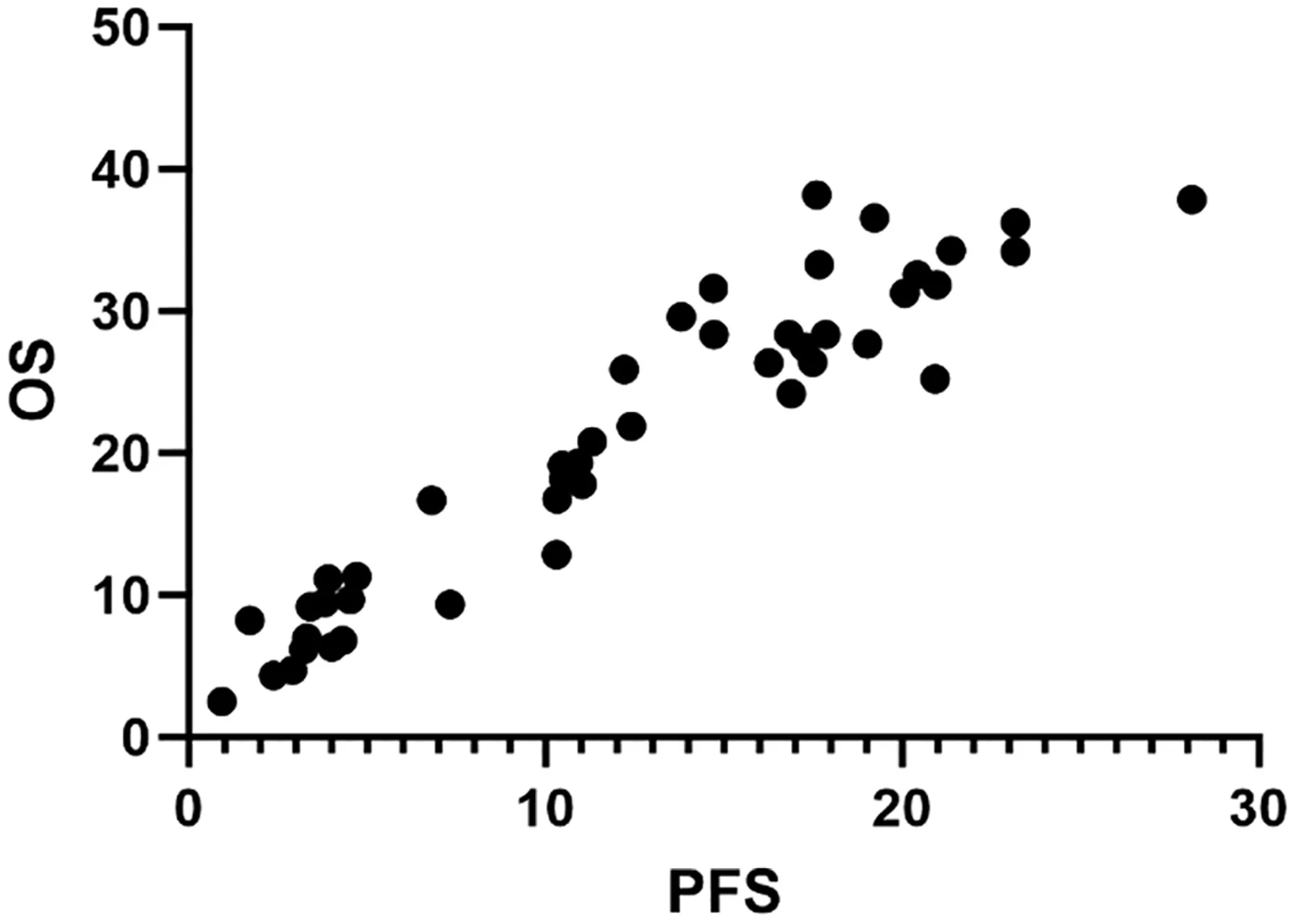
Figure of PFS and OS related scatter plot. Scatter plot demonstrating the correlation between progression-free survival (PFS) and overall survival (OS) in patients receiving TACE+PD-1 inhibitor+TKI therapy. Each dot represents 1 patient (n = 45); the x-axis shows PFS in months and the y-axis OS in months. The 2 endpoints were strongly and positively correlated (Pearson *R* = 0.95, *P* < .001), indicating that PFS tracks OS closely in this cohort and can serve as an early readout of benefit. OS = Overall Survival ,PFS = Progression-Free Survival, PD-1 = programmed death-1,TACE = transcatheter arterial chemoembolization, TKIs = tyrosine kinase inhibitors.

**Figure 5.**
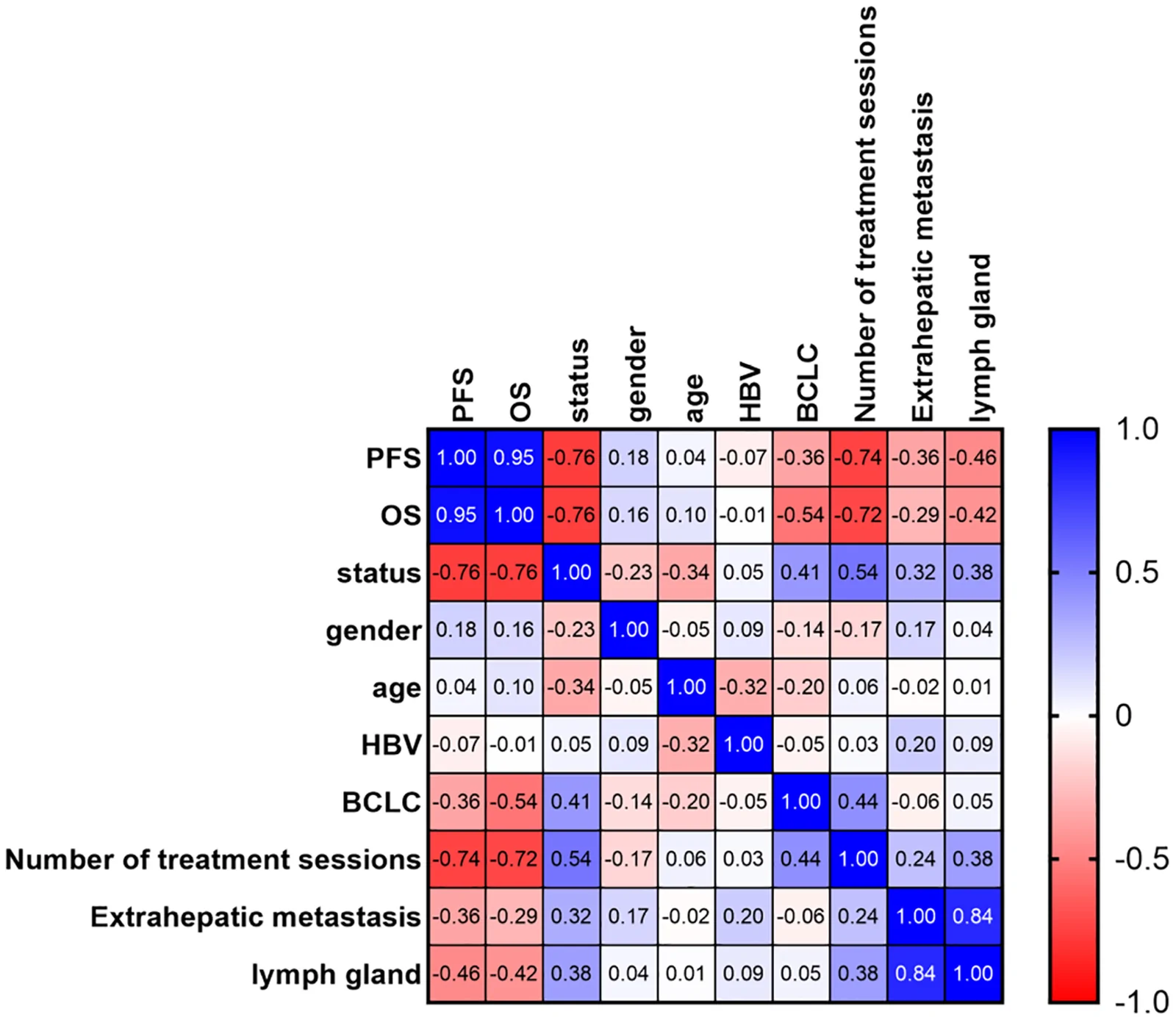
Analyzing the impact of multiple factors on PFS and OS outcome images. Heatmap of correlation analysis showing the linear association strength between multiple clinical variables, including PFS, OS, treatment line, node expression, BCLC staging, and extrahepatic metastasis. Cell values are Pearson correlation coefficients; blue denotes positive and red negative correlation, with color intensity proportional to the absolute value of *r*. Clinically, the map identifies which routinely available variables carry prognostic weight: the number of prior treatment lines (*r* = −0.74 for PFS) and lymph node involvement (r = −0.46 for PFS) show the strongest inverse associations with survival, whereas age, sex, and HBV status show none. It is therefore intended as a visual screen of candidate prognostic variables for patient selection, not as a test of treatment effect, and the coefficients denote association rather than causation. BCLC = Barcelona Clinic Liver Cancer , HBV = Hepatitis B Virus ,OS = overall survival ,PFS = progression-free survival.

#### 3.3.1. BCLC staging

By combining the Kaplan–Meier method with the Log-rank test, we further analyzed the impact of BCLC staging on PFS and OS. The analysis showed that the median PFS for the BCLC A, BCLC B, BCLC C, and BCLC D groups were 19.25 months (95% CI: 15.12–23.39), 16.51 months (95% CI: 12.41–20.61), 12.18 months (95% CI: 9.52–14.84), and 4.85 months (95% CI: 0.96–8.75), respectively (log-rank *P* = .016). The corresponding OS were 33.34 months (95% CI: 15.78–50.89), 29.22 months (95% CI: 26.11–32.33), 20.69 months (95% CI: 16.96–24.42), and 7.47 months (95% CI: 3.52–11.44), respectively (Table 4). Univariate Cox regression analysis revealed that compared with BCLC A (reference category), BCLC D was associated with significantly worse PFS (HR = 4.82, 95% CI: 1.45–16.02, *P* = .010) and OS (HR = 5.67, 95% CI: 1.58–20.35, *P* = .008). These results demonstrate that BCLC staging has a significant impact on survival outcomes, with patients at earlier BCLC stages showing more pronounced survival benefits.

**Table 4:** Survival outcomes by BCLC stage.

BCLC stage	n (%)	Median PFS, months (95% CI)	Median OS, months (95% CI)	HR for PFS (95% CI)	HR for OS (95% CI)	*P* value
A (reference)	3 (6.7)	19.25 (15.12−23.39)	33.34 (15.78–50.89)	1.00	1.00	–
B	5 (11.1)	16.51 (12.41–20.61)	29.22 (26.11–32.33)	Not estimated	Not estimated	–
C	32 (71.1)	12.18 (9.52–14.84)	20.69 (16.96–24.42)	Not estimated	Not estimated	–
D	5 (11.1)	4.85 (0.96–8.75)	7.47 (3.52–11.44)	4.82 (1.45–16.02)	5.67 (1.58–20.35)	.010 (PFS); .008 (OS)
Overall	45 (100)	–	–	–	–	.016 (log–rank, PFS)

HRs are from univariate Cox regression with BCLC A as the reference category; HRs for stages B and C were not estimated because of the small number of events. *P* values are 2-sided and reported to 3 decimals; values below .001 are reported as <.001.

BCLC = Barcelona Clinic Liver Cancer, CI = confidence interval, HR = hazard ratio, OS = overall survival, PFS = progression-free survival.

#### 3.3.2. Treatment line

The results of TACE + TKIs + PD-1 inhibitor therapy showed that both PFS and OS statistically significantly differed among different treatment lines (log-rank *P* < .001 for both). The median PFS for first-, second-, and third-line treatments were 17.15 months (95% CI: 14.81–19.48), 12.59 months (95% CI: 9.30–15.87), and 3.19 months (95% CI: 2.44–3.95), respectively. The corresponding OS values were 28.23 months (95% CI: 24.96–31.51), 21.54 months (95% CI: 16.34–26.75), and 7.21 months (95% CI: 5.46–8.98), respectively (Table 5). Cox regression analysis demonstrated that compared with first-line treatment (reference category), third-line treatment was associated with significantly worse PFS (HR = 6.28, 95% CI: 2.87–13.74, *P* < .001) and OS (HR = 5.41, 95% CI: 2.35–12.45, *P* < .001). These results suggested that the combination of TACE + TKIs + PD-1 inhibitor in the first-line treatment showed more significant survival benefits than that in the third-line treatment.

**Table 5:** Survival outcomes by treatment line.

Treatment line	n (%)	Median PFS, months (95% CI)	Median OS, months (95% CI)	HR for PFS (95% CI)	HR for OS (95% CI)	*P* value
First-line (reference)	20 (44.4)	17.15 (14.81–19.48)	28.23 (24.96–31.51)	1.00	1.00	–
Second-line	13 (28.9)	12.59 (9.30–15.87)	21.54 (16.34–26.75)	Not estimated	Not estimated	–
Third-line	12 (26.7)	3.19 (2.44–3.95)	7.21 (5.46–8.98)	6.28 (2.87–13.74)	5.41 (2.35–12.45)	<.001 (PFS and OS)
Overall	45 (100)	–	–	–	–	<.001 (log–rank, PFS and OS)

Treatment line is defined by the number of prior systemic regimens (first line, none; second line, one; third line, 2 or more). HRs are from univariate Cox regression with first-line treatment as the reference category. *P* values are 2-sided; values below .001 are reported as < .001.

CI = confidence interval, HR = hazard ratio, OS = overall survival, PFS = progression-free survival.

#### 3.3.3. Extrahepatic metastasis

Extrahepatic metastasis significantly impacted PFS and OS. The median PFS for patients with and without extrahepatic metastasis were 5.14 months (95% CI: 3.03–7.25) and 15.56 months (95% CI: 13.37–17.75), respectively (log-rank *P* < .001). The corresponding OS values were 9.68 months (95% CI: 5.95–13.42) and 26.13 months (95% CI: 23.11–29.16), respectively (log-rank *P* < .001) (Table 6). Cox regression analysis showed that the presence of extrahepatic metastasis (reference category: absent) was associated with significantly worse PFS (HR = 3.45, 95% CI: 1.72–6.92, *P* < .001) and OS (HR = 4.12, 95% CI: 1.98–8.57, *P* < .001). These findings suggest that extrahepatic metastasis is a crucial factor influencing the survival benefits of patients treated with TACE + TKIs + PD-1 inhibitor therapy.

**Table 6:** Impact of extrahepatic metastasis on survival.

Extrahepatic metastasis	n (%)	Median PFS, months (95% CI)	Median OS, months (95% CI)	HR for PFS (95% CI)	HR for OS (95% CI)	*P* value
Absent (reference)	31 (68.9)	15.56 (13.37–17.75)	26.13 (23.11–29.16)	1.00	1.00	–
Present	14 (31.1)	5.14 (3.03–7.25)	9.68 (5.95–13.42)	3.45 (1.72–6.92)	4.12 (1.98–8.57)	<.001 (PFS and OS)

HRs are from univariate Cox regression with absence of extrahepatic metastasis as the reference category.

Log-rank *P* < .001 for both PFS and OS. *P* values are 2-sided; values below .001 are reported as <.001.

CI = confidence interval, HR = hazard ratio, OS = overall survival, PFS = progression-free survival.

#### 3.3.4. Node expression

Node expression status also significantly affected treatment outcomes. The median PFS for patients with positive and negative node expression were 5.14 months (95% CI: 2.69–7.60) and 9.96 months (95% CI: 5.53–14.40), respectively (log-rank *P* < .001). The corresponding OS values were 14.92 months (95% CI: 12.68–17.17) and 25.03 months (95% CI: 21.80–28.27), respectively (log-rank *P* < .001) (Table 7). Cox regression analysis revealed that positive node expression (reference category: negative) was associated with worse PFS (HR = 2.89, 95% CI: 1.38–6.05, *P* = .005) and OS (HR = 3.24, 95% CI: 1.52–6.91, *P* = .002).

**Table 7:** Impact of node expression on survival.

Node expression	n (%)	Median PFS, months (95% CI)	Median OS, months (95% CI)	HR for PFS (95% CI)	HR for OS (95% CI)	*P* value
Negative (reference)	33 (73.3)	9.96 (5.53–14.40)	25.03 (21.80–28.27)	1.00	1.00	–
Positive	12 (26.7)	5.14 (2.69–7.60)	14.92 (12.68–17.17)	2.89 (1.38–6.05)	3.24 (1.52–6.91)	.005 (PFS); .002 (OS)

HRs are from univariate Cox regression with negative node expression as the reference category. Log-rank *P* < .001 for both PFS and OS.

*P* values are 2–sided; values below .001 are reported as <.001.

CI = confidence interval, HR = hazard ratio, OS = overall survival, PFS = progression-free survival. Node expression denotes lymph node involvement on baseline imaging.

### 3.4. Adverse events

The three most common AEs observed during TACE + TKIs + PD-1 inhibitor treatment were increased transaminase levels, tiredness, and nausea and vomiting. Although some severe AEs of Level 3 or 4 were observed, most were Level 1 or 2; no fatal AEs were reported. Level 3 and 4 AEs included 2 cases of tiredness (4.44%), 3 cases of abdominal pain (6.67%), 3 cases of nausea and vomiting (6.67%), 2 cases of diarrhea (4.44%), 2 cases of hypertension (4.44%), and 4 cases of increased transaminase levels (8.89%) (Table 8).

**Table 8:** Treatment-related adverse events (n = 45).

Adverse event	Grade 1–2, n (%)	Grade 3–4, n (%)	Grade 5, n (%)	Total, n (%)
Increased transaminase	18 (40.0)	4 (8.9)	0 (0)	22 (48.9)
Tiredness	15 (33.3)	2 (4.4)	0 (0)	17 (37.8)
Nausea and vomiting	12 (26.7)	3 (6.7)	0 (0)	15 (33.3)
Abdominal pain	10 (22.2)	3 (6.7)	0 (0)	13 (28.9)
Diarrhoea	8 (17.8)	2 (4.4)	0 (0)	10 (22.2)
Hypertension	6 (13.3)	2 (4.4)	0 (0)	8 (17.8)

Adverse events were graded according to the Common Terminology Criteria for Adverse Events (CTCAE) version 5.0. Percentages are based on the 45 patients in the final analysis; a patient may have experienced more than 1 event, so columns do not sum to 100. No grade 5 event occurred.

## 4. Discussion

The present retrospective study evaluated the efficacy and safety of TACE combined with TKIs and PD-1 inhibitors in patients with advanced unresectable HCC. Our results demonstrated a median PFS of 12.4 months and median OS of 24.2 months, with an ORR of 44.4% and DCR of 91.1%, suggesting that this triple combination therapy provides meaningful clinical benefits in this patient population. Because the design was retrospective, single-arm, and single-center, these figures describe what was observed in a selected cohort rather than a treatment effect, and the analysis should be regarded as exploratory and hypothesis-generating.

### 4.1. Comparison with previous studies

A retrospective study of 120 patients with unresectable HCC involving propensity score matching reported that TACE + TKIs + ICIs vs TKIs + ICIs groups had a median PFS of 8.4 vs 6.6 months (*P* = .115) and median OS of 26.9 vs 24.2 months (*P* = .670).^[17]^ PFS subgroup analysis showed that triple therapy had a more significant benefit (*P* = .015) in patients without prior local treatment. The median PFS in this study (12.4 months) was higher than the 8.4 months reported by Pan et al. This difference may be attributed to several factors: the inclusion of more BCLC A/B stage patients in this study (17.8% vs <10% in their study) and a higher proportion of first-line treatment (44.4% vs 35%). Additionally, Pan’s study included patients who failed second-line treatment, which likely contributed to a greater tumor burden in their cohort.

A phase II trial involving multiple centers and a single treatment arm reported a 35.6% ORR, alongside a median PFS of 10.5 months.^[18]^ Our study demonstrated a comparable ORR (44.4%) and slightly longer PFS (12.4 months), further supporting the efficacy of TACE + TKIs + ICIs therapy in patients in the first-line setting. From 2012 to 2019, the clinical advantages of combining TACE with targeted therapy consistently improved.^[19]^ Several studies showed that TACE and targeted treatment considerably increased OS by over 50%.^[20]^

The Phase III IMbrave150 study demonstrated that atezolizumab and bevacizumab combination therapy for first-line treatment of advanced HCC reduced the risk of death by 42% (HR = 0.58; 95% CI: 0.42–0.79; *P* = .0006) and disease progression by 41% (HR = 0.59; 95% CI: 0.47–0.76; *P* < .0001) compared with sorafenib. The median OS (24.2 months) of the triple therapy in this study was numerically superior to the sorafenib group (10.7 months) in the IMbrave150 trial and even exceeded that of its combination therapy group (19.2 months). This potential advantage may be explained by the synergistic effect between TACE’s local control function and immunotherapy/targeted therapy: TACE induces tumor antigen release, enhancing the immune response to PD-1 inhibitors; meanwhile, TKIs inhibit the VEGF pathway, reversing the hypoxic microenvironment post-TACE and reducing programmed death-ligand 1upregulation.

### 4.2. Impact of treatment timing

This study analyzed the timing of treatment selection for TACE + TKIs + ICIs. The research results indicate that, in the treatment of advanced HCC, patients receiving first-line therapy with TACE + TKIs + ICIs demonstrated significantly superior PFS (17.15 months) and OS (28.23 months) compared to second-line therapy with PFS (12.59 months) and OS (21.54 months). Meanwhile, third-line therapy yielded the poorest outcomes, with PFS (3.19 months) and OS (7.21 months). The Cox regression analysis confirmed this trend, showing that third-line treatment was associated with a 6.28-fold increased risk of disease progression (HR = 6.28, 95% CI: 2.87–13.74) compared to first-line treatment.

The markedly reduced efficacy observed in the third-line setting may be attributed to several biological mechanisms. First, repeated treatments likely promote enhanced tumor heterogeneity and expansion of drug-resistant clones. Second, prolonged exposure to prior therapies may lead to immune exhaustion, characterized by T-cell dysfunction and reduced responsiveness to checkpoint inhibition. This aligns with the CheckMate040 study, where nivolumab demonstrated an ORR of merely 14.3% in third-line treatment, highlighting the limitations of late-stage intervention.^[21]^ These findings collectively support the concept of early intervention with TACE + TKIs + ICIs to maximize clinical benefits.

### 4.3. Prognostic factors

This study also conducted analyses on multiple prognostic factors, evaluating the impact of the TACE + TKIs + ICIs treatment regimen on PFS and OS. The results indicate that the factor with the greatest influence on treatment outcomes is treatment line (*r* = −0.739), followed by node expression (*r* = −0.462), extrahepatic metastasis (*r* = −0.360), and BCLC staging (*r* = −0.357), while the effects of age, gender, and HBV status on treatment results were not statistically significant. The pattern is more informative than the individual coefficients: outcome was governed by where the disease sat in its treatment trajectory and by the extent of tumor spread, not by host factors such as age, sex, or viral status. This suggests that the achievable benefit is set mainly by residual tumor burden and by preserved immune competence at the time the regimen is introduced, and it argues for positioning triple therapy earlier in the sequence rather than reserving it for salvage.

The impact of BCLC staging on survival outcomes warrants particular attention. Patients with BCLC stage D have an extremely short survival period (median OS 7.47 months), with Cox regression demonstrating a 5.67–fold increased risk of death compared to BCLC A patients (HR = 5.67, 95% CI: 1.58–20.35). This finding may be explained by poor tolerance to systemic therapy caused by liver decompensation or distant metastasis. In contrast, BCLC stage A patients retain better liver function, allowing TACE to more thoroughly embolize tumor blood vessels, resulting in more significant synergistic effects when combined with immunotherapy and targeted therapy.

Regarding extrahepatic metastasis, our analysis revealed that patients with extrahepatic spread had significantly worse outcomes (median PFS: 5.14 vs 15.56 months; HR = 3.45). This finding is clinically important, as it suggests that the benefits of triple therapy are most pronounced in patients with disease confined to the liver, where TACE can achieve optimal local control.

Similarly, positive node expression was identified as an independent adverse prognostic factor (HR = 2.89 for PFS, HR = 3.24 for OS). The presence of lymph node involvement may indicate more aggressive tumor biology and reduced responsiveness to locoregional therapy.

### 4.4. Safety profile

The present study found no fatal adverse events in combination therapy cases, and the main AEs were at Levels 1 to 2, suggesting that the combination therapy was well-tolerated. The most common Grade 3 to 4 AEs were increased transaminase levels (8.89%), abdominal pain (6.67%), and nausea/vomiting (6.67%), which are consistent with the known safety profiles of the individual treatment components. These findings are reassuring and suggest that the triple combination does not result in unexpected or cumulative toxicities.

### 4.5. Study limitations

This study has several important limitations that should be acknowledged. First, the single-center, single-arm retrospective design precludes definitive causal inference and makes it impossible to exclude selection bias. Patients enrolled in this study may have had better baseline performance status compared to the general population with advanced HCC.

Second, most of the enrolled patients had BCLC C (71.1%), leading to potential bias in the overall data analysis. The limited number of patients in BCLC A (n = 3) and BCLC B (n = 5) groups restricts the generalizability of subgroup findings to earlier-stage disease.

Third, various TACE schemes and combinations of TKIs and PD-1 inhibitors were applied in this study, introducing heterogeneity that may affect treatment outcomes. The lack of standardization in drug regimens makes it difficult to determine the optimal combination.

Fourth, this study did not perform multivariate Cox regression analysis to adjust for potential confounders such as alpha-fetoprotein levels>400 ng/m, major vascular invasion, and tumor burden. This limitation may lead to overestimation of treatment efficacy.

Fifth, the relatively small sample size (n = 45) limits statistical power, particularly for subgroup analyses. No formal sample size calculation was performed prior to study initiation.

Finally, the median follow-up duration was not reported, and longer follow-up is needed to assess the durability of treatment responses and long-term survival outcomes.

### 4.6. Future directions

Based on our findings, several areas warrant further investigation. Prospective, randomized controlled trials comparing TACE + TKIs + PD-1 inhibitors with standard-of-care treatments are needed to confirm these results. Additionally, biomarker studies to identify patients most likely to benefit from triple therapy would help optimize patient selection. Finally, standardization of treatment protocols, including TACE techniques and drug combinations, would facilitate comparison across studies. Until such data are available, TACE + TKIs + PD-1 inhibitors should not be adopted as standard care outside clinical trials or multidisciplinary team decisions.

## 5. Conclusion

In summary, this exploratory retrospective analysis found that TACE + TKIs + ICIs therapy was associated with encouraging efficacy in patients with advanced HCC receiving first-line treatment, those with BCLC stages A to B, negative node expression, and no extrahepatic metastasis. Conversely, patients receiving third-line treatment, those with BCLC stage C to D, or those with extrahepatic metastasis showed diminished treatment benefits. Personalized treatment selection based on these prognostic factors is needed in clinical practice to optimize efficacy and quality of life. Prospective, randomized studies are warranted to validate these findings. Given the single-arm retrospective design and the limited sample size, these observations are hypothesis-generating and should not be used to guide routine clinical practice until confirmed in larger prospective studies.

## Acknowledgments

This research was supported by the Jiangxi Provincial Health Science and Technology Program (SKJP220201578).

## Author contributions

**Project administration:** Guohui Xu.

**Software:** Guohui Xu, Xiaojun Li.

**Data curation:** Jianlong Zhang, Shilan Fan, Xiao Wu.

**Formal analysis:** Jianlong Zhang.

**Supervision:** Xiaojun Li, Cuncai Zhou.

**Methodology:** Shilan Fan.

**Conceptualization:** Xiao Wu.

**Resources:** Cuncai Zhou.

**Writing – original draft:** Guohui Xu.

**Writing – review & editing:** Cuncai Zhou.
